# Broadband EMI Shielding Performance in Optically Transparent Flexible In_2_O_3_/Ag/In_2_O_3_ Thin Film Structures

**DOI:** 10.3390/ma18235393

**Published:** 2025-11-29

**Authors:** Anton S. Voronin, Sergey V. Nedelin, Nikita A. Zolotovsky, Igor A. Tambasov, Mstislav O. Makeev, Pavel A. Mikhalev, Bogdan A. Parshin, Evgenia L. Buryanskaya, Mikhail M. Simunin, Ilya V. Govorun, Ivan V. Podshivalov, Il`ya I. Bril`, Mikhail K. Khodzitskiy, Stas V. Khartov

**Affiliations:** 1 Regional Educational and Scientific Center “Security”, Bauman Moscow State Technical University, 105005 Moscow, Russia; pamikhalev@bmstu.ru (P.A.M.); parshbgal@bmstu.ru (B.A.P.); buryanskayael@bmstu.ru (E.L.B.); michanel@mail.ru (M.M.S.); ellaijiah@gmail.com (I.I.B.); 2 Department of Molecular Electronics, Federal Research Center «Krasnoyarsk Scientific Center», Siberia Branch, Russian Academy of Sciences (FRC KSC SB RAS), 660036 Krasnoyarsk, Russia; 3 School of Engineering and Construction, Siberian Federal University, 660041 Krasnoyarsk, Russia; 4 School of Engineering Physics and Radio Electronics, Siberian Federal University, 660041 Krasnoyarsk, Russia; s.v.nedelin@mail.ru (S.V.N.); nikitazolotovskiy@mail.ru (N.A.Z.); podshivalov.ivan@gmail.com (I.V.P.); 5 LLC Research and Production Company «Spectehnauka», 660043 Krasnoyarsk, Russia; tambasov_igor@mail.ru; 6 Laboratory of Photonics of Molecular Systems, Kirensky Institute of Physics, Siberian Branch, Russian Academy of Sciences, 660036 Krasnoyarsk, Russia; 7 School of Non-Ferrous Metals and Materials Science, Siberian Federal University, 660041 Krasnoyarsk, Russia; 8 Laboratory of Electrodynamics and Microwave Electronics, Kirensky Institute of Physics, Siberian Branch, Russian Academy of Sciences, 660036 Krasnoyarsk, Russia; govorun-ilya@mail.ru; 9 Institute of Informatics and Telecommunication, Siberian State University of Science and Technology, 660037 Krasnoyarsk, Russia; 10 LLC “Terahertz Photonics”, 191167 Saint-Petersburgh, Russia; khodzitskiy@yandex.ru

**Keywords:** thin films, oxide/metal/oxide structures, EMI shielding, THz-TDS, In_2_O_3_

## Abstract

Transparent conductive electrodes that combine flexibility with effective electromagnetic interference (EMI) shielding are important for next-gen flexible electronics and 5G/6G communication devices. Achieving high optical transparency, low sheet resistance, and broadband shielding performance remains a sophisticated task. This work demonstrates a solution: the synthesis and comprehensive characterization of flexible In_2_O_3_/Ag/In_2_O_3_ (IAI) structures on polyethylene terephthalate substrates. The optimized structure with a 13.2 ± 1.1 nm silver interlayer achieves an incredible combination of properties: high optical transmittance (82.59% at 500 nm), low sheet resistance (6.4 ± 0.8 Ω/sq), and insignificant optical haze (1.04%). Broadband EMI shielding measurements from 10 MHz to 1 THz reveal a uniform shielding effectiveness of 25–30 dB across band from radiowave to terahertz. The IAI structures also show outstanding mechanical resilience, maintaining their electrical and shielding performance under repeated bending. This unique set of attributes positions IAI thin films as a prospective material for transparent EMI shielding in advanced telecommunications and flexible optoelectronics.

## 1. Introduction

The development of optically transparent screens is an important task at the intersection of applied electrodynamics and materials science. Its importance is driven by the needs of telecommunications, metrology, and energy-efficient construction [1]. The combination of high optical transparency and high shielding efficiency (dependent on sheet resistance) is a technological paradox that limits the creation of such structures. The key development challenge is creation of material with optical transparency of at least 80% and a shielding efficiency greater than 30 dB over the widest possible band. The main solutions of this problem are metal meshes [2,3], transparent conducting oxides (TCO) [4,5] and metal nanowires [6,7,8]. All these materials demonstrate fundamental physical and chemical limitations. Metal meshes have a frequency-dependent shielding efficiency due to diffraction effects on the perforated structure. They are described by the Kontorovich model [9] and generally have low efficiency in the *K* and *K_a_* bands, which are particularly reserved for 5G frequencies. Transparent conducting oxides face a fundamental trade-off between charge carrier concentration and optical transparency, described by the Drude relation [10]. This limits the possibility of obtaining TCO with resistances below 10 Ω/sq. The ceramic nature of TCO limits the use of flexible substrates. Metallic nanowires create percolation networks with high optical scattering [11], as a result, sheet resistance values acceptable for shielding applications are achieved only with low optical characteristics. Their corrosion under atmospheric conditions is an additional limiting factor [12], which can be solved by passivating the nanowires, for example, rGO [13], PEDOT:PSS [14] or MXenes [15].

One of the most promising classes of materials for use as broadband radio wave shields are thin-film oxide/metal/oxide (OMO) structures [16,17,18]. The metal layers that are mainly considered are Ag [19,20], Cu [21] and Au [22]. ITO [22,23], AZO [24], GZO [25], TiO_2_ [26] and others are promising as top and bottom oxide layers. A wide range of oxide layers is used for OMO structures. Wide possibilities in the choice of materials for OMO structures allow tuning functional parameters such as sheet resistance, optical transmittance, work function, etc., for each specific application. OMO structures have low sheet resistance of less than 10 Ω/sq with optical transparency of more than 80% and minimal scattering. In addition, OMO structures demonstrate high chemical stability and compatibility with flexible substrates, which makes them promising candidates for use in optoelectronic devices, transparent and flexible electronics, and 5G/6G communication systems. For communication applications, OMO structures are promising as broadband shields effective from the VHF to the EHF range [27], optically transparent absorbers [28], and optically transparent antennas [29].

In this study, we performed ultra-broadband measurements of the shielding properties of In_2_O_3_/Ag/In_2_O_3_ thin-film structures with variable silver layer thickness on flexible polyethylene terephthalate (PET) substrates in the frequency range from 10 MHz to 1 THz. The choice of this structure for studying the broadband shielding properties was justified by several advantages. Firstly, silver (Ag) has the lowest resistivity among all metals, which is a key factor in achieving low sheet resistance and high shielding efficiency. Secondly, indium oxide (In_2_O_3_) was chosen as the top and bottom layers due to its high optical transparency in the visible range. The top In_2_O_3_ layer acts as an antireflection coating, reducing optical reflection losses. The bottom In_2_O_3_ layer serves as the basis for the formation of a continuous and smooth silver layer of thin thickness. In_2_O_3_ is chemically inert and mechanically rigid. It forms a dense barrier that effectively protects the intermediate silver layer from oxygen and moisture diffusion, as well as from mechanical damage during bending. This is important for flexible electronics applications and advanced shielding materials. Studying the In_2_O_3_/Ag/In_2_O_3_ structure and demonstrating its advantages over traditional materials (TCO, metal meshes) and alternative OMO structures is crucial for the implementation of optically transparent and flexible shielding coatings in next-generation communication systems (5G/6G).

## 2. Materials and Methods

### 2.1. Magnetron Sputtering IAI Structures

In_2_O_3_/Ag/In_2_O_3_ structures (IAI structures) were formed by sequential pulsed DC magnetron sputtering. A top and bottom layer consisting of In_2_O_3_ were deposited on a 50 μm-thick PET substrate (Hi-Fi Industrial Film Ltd., Stevenage, Hertfordshire, UK). Ag was used as the central layer. We consider symmetric IAI structures with three different Ag layer thicknesses. The estimated Ag deposition rate was 1.1 Å/s. We formed three types of IAI-1, IAI-2, and IAI-3 structures. Ag sputtering was carried out on a silver target (99.99% purity, Kurt J. Lesker Company GmbH, Dresden, Germany) in an argon atmosphere at a power of 50 W and a residual pressure of 4.4 × 10^−3^ Torr. In_2_O_3_ was deposited reactively from an indium metal target (99.99% purity, Kurt J. Lesker Company GmbH, Dresden, Germany) at a power of 100 W in an argon/oxygen atmosphere with a ratio of 79%/21%, respectively, and a residual pressure of 4.4 × 10^−3^ Torr. The estimated sputtering rate of indium oxide is 1.7 Å/s. One of the advantages was the choice of reactive sputtering of indium in an oxygen-containing environment. Metallic targets (In) offer several advantages over ceramic targets (ITO): they are less prone to cracking, and the sputtering process is more stable. Furthermore, metal targets are often cheaper to manufacture than high-quality stoichiometric ceramic targets. Ceramic targets (In_2_O_3_ or ITO) are brittle, prone to cracking due to overheating (they have low thermal conductivity), and their production with ideal stoichiometry is more difficult and expensive.

To ensure the reproducibility of the process, the following critical parameters were maintained at a constant for all depositions. The target-to-substrate distance was fixed at 100 mm. The sputtering pressure was actively controlled at 4.4 × 10^−3^ Torr with a stability of ±0.1 × 10^−3^ Torr. The substrate holder was not actively heated, and the deposition was carried out at room temperature (~25 °C), which is essential for preventing thermal deformation of the flexible PET substrate. The thickness of each layer was determined using a quartz resonator.

### 2.2. Scanning and Transmission Electron Microscopy, EDX, XRD Analysis and Atomic Force Microscopy of IAI Structures

The morphological properties of In_2_O_3_ films were studied using scanning electron microscopy (SEM) on an S5500 and SU3500 microscopes (Hitachi, Tokyo, Japan). In-plane elemental analysis was performed using an XFlash 430 Energy-Dispersive spectrometer (EDX, Bruker, Billerica, MA, USA).

Cross-sections for transmission and scanning electron microscopy (TEM) were prepared using the lift-out method with a focused ion beam in a Scios SEM (FEI Company, Hillsboro, OR, USA). To prevent surface damage, a protective Pt layer of approximately 1 µm thickness was deposited on the surface of the samples before cross-section preparation. Cross-sectional studies were performed on an Osiris microscope (Thermo Fisher Scientific, Waltham, MA, USA) equipped with a high-angle annular dark-field detector (E.A. Fischione Instruments, Inc., 9003 Corporate Circle, Export, PA, USA) and a Super X-ray energy-dispersive spectrometer (ChemiSTEM, Bruker, Billerica, MA, USA).

The XRD data of IAI structures for Rietveld analysis were collected at room temperature with a Haoyuan DX-2700BH (Haoyuan, Dandong, China) powder diffractometer with Cu-Kα radiation and a linear detector. The step size of 2θ was 0.01°, and the counting time was 0.2 s per step. Therefore, these structures were taken as the starting model for Rietveld refinement, which was performed using TOPAS 4.

The surface roughness of IAI structures was studied by atomic force microscopy (AFM, Ntegra Prima (NT-MDT SI, Zelenograd, Russia)), the radius of curvature of the probe was 10 nm.

### 2.3. Optoelectronic Properties

Optical transmission spectra of the IAI structures in the 300–2500 nm range were obtained using a UV-3600i Plus spectrophotometer with an integrating sphere (Shimadzu, Kyoto, Japan).

According to ASTM D1003-21 [30], The haze parameter is determined according to the following formula:

(1) $$Haze=\left(\frac{T_{d}}{T_{t}}\right)\cdot 100\%$$

T_d_—diffuse transmission T_t_—total transmission.

The sheet resistance of IAI structures was measured by the four-probe method using a JG ST2258 four-point probe station (Suzhou Jingge Electronics Co., Suzhou, China) and a JG ST2558-F01 four-probe head (Suzhou Jingge Electronics Co., Suzhou, China).

The study of temperature dependences of sheet resistance of IAI structures, in the temperature interval 25–200 °C was carried out on an automated station STN 300.600.3 Omega (LLC Research and Production Company «Spectehnauka», Krasnoyarsk, Russia).

### 2.4. Measurements the Shielding Properties IAI Structures in the Range of 0.01–40 GHz

The scattering parameters S_11_ and S_21_ were measured using a 16.00/6.95 mm diameter coaxial cell (Type II, 50 Ω) with a sample slot (19.00/4.8 mm). The test setup consisted of a FieldFox N9916A vector network analyzer (Keysight Technologies, Santa Rosa, CA, USA), coaxial assemblies, and a cell.

Spectroscopy in the 18–40 GHz range was also performed using the waveguide method. Measurements were made in the K-band (18–26.5 GHz) and K_a_-band (26.5–40 GHz) ranges. The waveguide windows had a rectangular cross-section with dimensions of 4.3 × 10.65 mm for the K-band and 3.55 × 7.1 mm for the Ka-band. The measurements were performed on an R&S ZVA 50 vector network analyzer (Rohde & Schwarz GmbH & Co. KG, Munich, Germany).

### 2.5. THz Time-Domain Spectroscopy (THz-TDS)

The spectral properties of IAI structures in the terahertz frequency range were studied using terahertz time-domain spectroscopy (THz-TDS). The measurements were performed with a spectrometer featuring a dynamic range of over 70 dB at 0.4 THz and a spectral range of 0.1–1 THz. The system provides real-time data collection at a rate of 10 spectra/s with a spectral resolution of up to 1 GHz. Photoconductive antennas were employed as the THz emitter and detector. The sample was placed in the focal zone between the parabolic mirrors of the THz-TDS spectrometer. For each sample, three THz waveforms were recorded: a reference signal through empty space (air), a signal through the bare PET substrate (PET), and a signal through the IAI structure on the substrate (IAI + PET). To obtain the transmission spectra, the recorded time-domain signals were processed using a time-domain window to isolate the main transmitted pulse and exclude subsequent signals caused by multiple internal reflections within the dielectric substrate. This approach minimizes the influence of interference effects on the resulting spectra.

### 2.6. Mechanical and Chemical Stability Tests

To determine the effect of bending on electrical and shielding parameters, we made of single-outward bending tests with the different radii: 100, 10, 5, 2, 1, and 0.5 mm. The corresponding tool with control radius was manufactured using 3D printing. The bending time was 3 s. To characterize the sheet resistance of IAI-3 structures to cyclic loading, we subjected them to 1000 bending cycles. Cyclic bending tests were conducted at a radius of 5 mm. The number of bending cycles was 1000. For testing, we selected the IAI-3 structure with the best parameters. Three samples were used for each test, and the data were averaged.

Chemical stability of IAI structures to external conditions was studied in two modes. The first mode is a two-week exposure of IAI structures at 100% humidity and room temperature, which was 21 °C. The second mode is a hundred-hour exposure of IAI structures at a temperature of 75 °C and a humidity of 100%. The experimental procedure was as follows: Distilled water was poured into the bottom of the Petri dish to maintain 100% humidity. The test sample was then added to the Petri dish and autoclaved according to the above test protocols. The sheet resistance of the coating was used as the evaluation criterion.

## 3. Results

### 3.1. Morphological Features of IAI Structures

**Figure 1:**
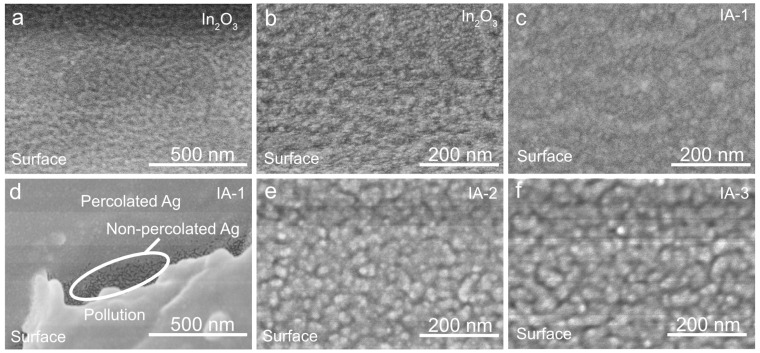
SEM image of underlayer In_2_O_3_ on PET (**a**,**b**) and IA-1 (**c**,**d**), IA-2 (**e**) and IA-3 (**f**).

The key role of scanning electron microscopy in the study of IAI structures is evaluating the filling factor and percolation properties of thin Ag films. Figure 1a,b shows SEM images of the In_2_O_3_ layer at different magnifications. Polycrystalline structure with a grain size in the 20–50 nm range is observed. The morphology of the In_2_O_3_ layer is important because it determines the morphology and roughness of the Ag layer deposited on it. To study the morphology of Ag films, we obtained structures without a top indium oxide layer, abbreviated as IA. The SEM image of the Ag IA-1 (Figure 1c) shows stable percolation between grains and a fine-grained structure. To minimize errors associated with image contrast, a defective region with local shading formed during deposition was highlighted. In this zone, an isolated Ag island has formed, devoid of percolation (Figure 1d). Stable percolation is also observed for Ag films IA-2 (Figure 1e) and IA-3 (Figure 1f). with an increase in film thickness leading to an increase in grain size. The contrast between the percolated and non-percolated regions confirms that all three types of Ag films have a quasi-continuous polycrystalline structure.

**Figure 2:**
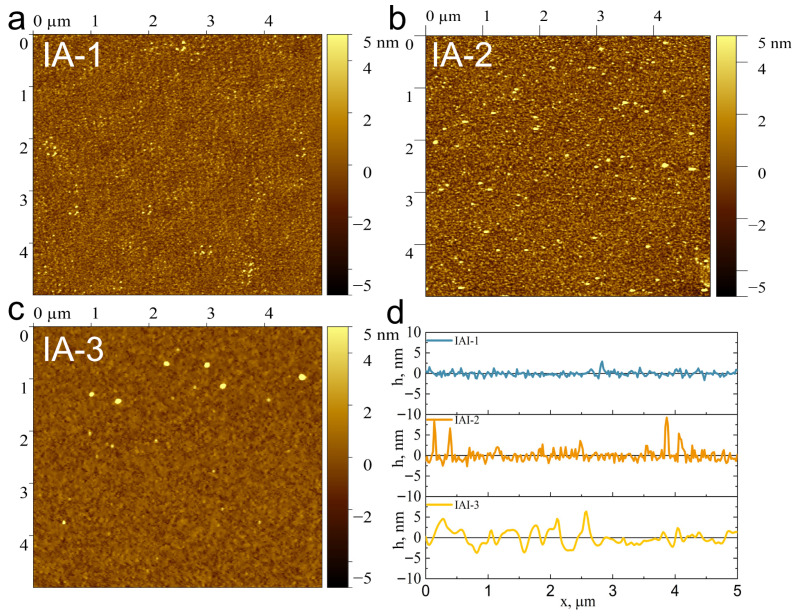
AFM images of IA-1 (**a**), IA-2 (**b**) and IA-3 (**c**) on Si substrate and their profiles (**d**).

The surface roughness of IA structures was studied using AFM (Figure 2a–c). The results show a trend: as the Ag layer thickness increases, the layer roughness increases. There are limitations associated with the curvature radius of the AFM probe, which is 10 nm, which is comparable to the grain size of the IA-1 film (Figure 1c). The roughness of the Ag layers on the indium oxide sublayer is 0.94 (IA-1), 1.79 (IA-2) and 1.94 nm (IA-3) (Figure 2d). The formation of thin metal films, including Ag, occurs via the island mechanism (Volmer–Weber), which determines the nonlinear dependence of roughness on thickness. The roughness reaches a maximum near the percolation threshold (4–7 nm), corresponding to the closure of isolated islands into a continuous film. In our work we deal with Ag films after the percolation threshold, and the dependence of the surface roughness on the thickness of the Ag film corresponds to the wetting regime [20].

**Figure 3:**
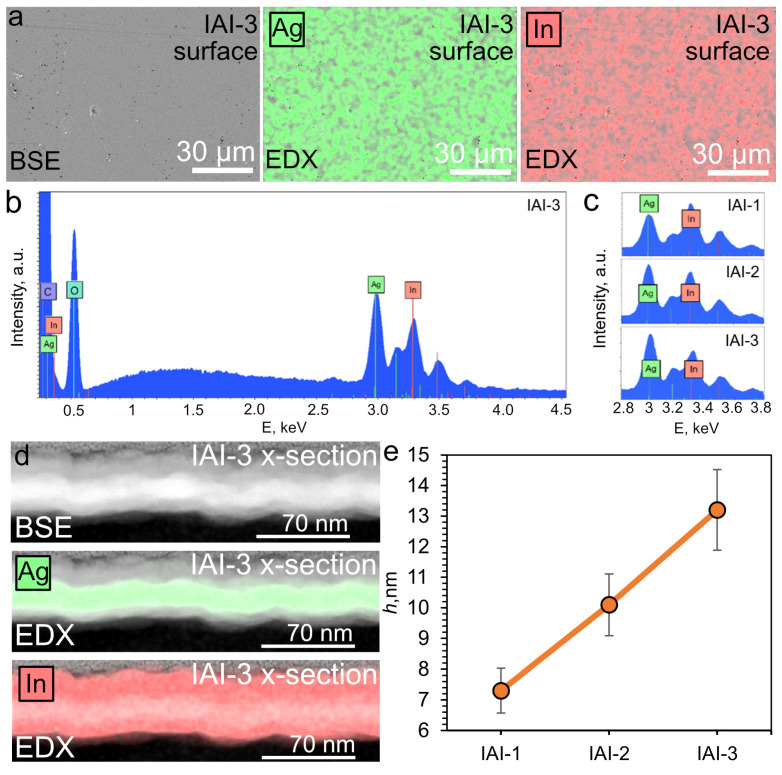
SEM images and EDX maps of IAI-3 structures in plane (**a**); EDX spectra of IAI structures in plane (**b**,**c**); cross-section of IAI-3 and their EDX images of Ag and In distribution (**d**); Thickness of the three studied IAI structures (**e**).

Elemental analysis can qualitatively assess the presence of a particular element in a material and the uniformity of its distribution across the sample area being studied. Figure 3a shows an SEM image of the surface of sample IAI-3 and elemental mapping for In (red) and Ag (green).

Figure 3b shows the EDX spectrum of the IAI-3 structure. The spectrum contains In peaks related to the L_α1_ series (3.286 keV), L_β1_ series (3.487 keV), L_β2_ series (3.712 keV). The Ag peaks belong to the L_α1_ series (2.983 keV) and L_β1_ series (3.150 keV). Figure 3c shows enlarged fragments of the spectra. The dependence is shown: with an increase in the thickness of the deposited Ag, the intensity ratio of the Lα1 In and Ag series changes. This is evidence of an increase in the amount of Ag in the studied structure. TEM and EDX scanning in the cross-sectional geometry of the IAI-3 sample are shown in Figure 3d,e. Based on the TEM results, we were able to estimate the thickness of each of the three layers. It is clear that the lower and upper indium oxide layers differ in thickness, creating a certain asymmetry. The Ag thicknesses in the IAI structures are shown in Figure 3e and are 7.3 ± 0.7, 10.1 ± 0.9, and 13.2 ± 1.1 nm.

To complement the electron microscopy data, we performed X-ray diffraction (XRD) analysis on the IAI structures (see Figure S1 in Supplementary Materials). The XRD patterns are dominated by a broad halo from the amorphous PET substrate. A distinct peak corresponding to the (111) plane of crystalline silver is observed for the IAI-3 structure, with a calculated crystallite size of approximately 9 nm using the Scherrer equation. No diffraction peaks from crystalline In_2_O_3_ were detected, confirming that both the top and bottom oxide layers are amorphous. This finding is consistent with the TEM results (Figure 3d,e) and is typical for oxide layers sputtered at room temperature onto flexible polymer substrates.

### 3.2. Optoelectronic Properties of IAI Structures

**Figure 4:**
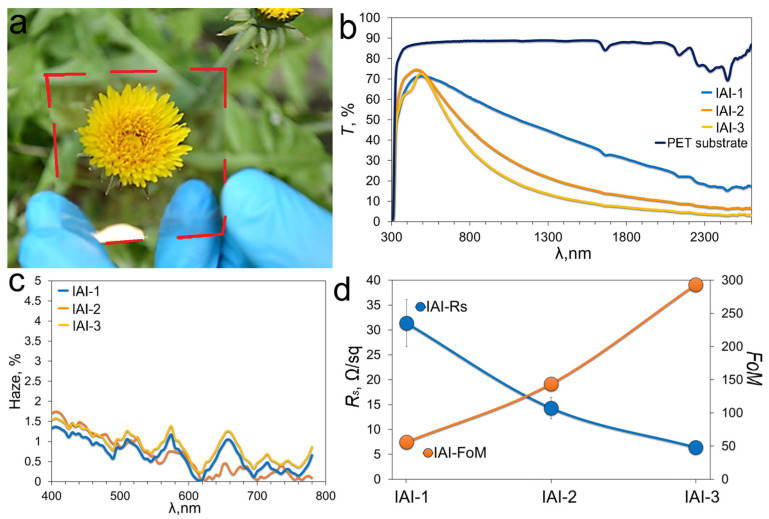
Photo of IAI-3 structure (**a**); transmittance (**b**) and haze (**c**) spectra of IAI in UV-Vis; sheet resistance and FoM of IAI structures with different Ag thickness (**d**). We indicated the values of the optoelectric parameters in Table 1, which is presented below in the text.

Figure 4a shows the IAI-3 structure. The transmittance of the IAI structures is shown in Figure 4b. The reference PET substrate has uniform transmittance in the visible range, with a value of 87.51% at a wavelength of 500 nm. In the near UV (300–400 nm), the transmittance decreases to zero due to strong absorption due to π→π* transitions. This determines the opacity of the IAI structures in the near UV. All three configurations of IAI structures show high transmittance in the visible region and strong suppression in the UV and IR ranges. Analysis of the spectra shows that the thickness of the Ag intermediate layer significantly affects both the transmittance and the spectral position of the transmittance maximum. A characteristic feature of metal films is a decrease in transparency with increasing thickness, caused mainly by light reflection at the metal-air interface, while the intrinsic absorption of the metal layer plays a secondary role. Deposition of indium oxide on top of the metal film allows for a significant reduction in reflection losses. They are used as an antireflection layer. The thinnest Ag interlayer (IAI-1, 7.3 ± 0.7 nm) demonstrates a wider transmission profile with a maximum transparency of ~71.54% at a wavelength of 495 nm, but the lowest losses in the IR range. While thicker Ag layers (IAI-3, 13.2 ± 1.1 nm) demonstrate a significant decrease in transmittance in the IR range, the maximum transmission lines at a wavelength of 486 nm and is ~72.62%. Oxide layers in IAI structures act as an antireflective coating. As a rule, their thickness determines the position of the maximum transmittance [31]. Transparent conductors based on Ag [32,33] and Cu [34] metal films have lower transmittance than OMO structures.

Figure 4c shows the haze spectra for IAI structures in the visible range, calculated according to Equation (1). All spectra have an identical trend, with a slight difference in the absolute value of the haze parameter.

The electric properties of IAI structures can be described using a model of three parallel-connected resistors: two high-resistance layer (In_2_O_3_) and one low-resistance layer (Ag). Thus, the sheet resistance of the IAI structure is determined by the resistance of the Ag layer. The sheet resistance of the Ag layer can be estimated based on the specific resistivity of silver, which is approximately ~1.6 × 10^−8^ Ω·m for bulk single-crystal material. However, since the resistivity of thin films differs from that of the bulk due to enhanced surface and grain boundary scattering, it should be recalculated accordingly using the Fuchs–Sondheimer approach [35]. Then the sheet resistance can be calculated according to R = ρ/d. Substituting the silver thicknesses of the IAI structures into the formula yields values of 8.0, 5.0 and 3.3 Ω/sq, respectively. In this work, the measurements were averaged over 20 points, and the resulting curve is presented in Figure S2 Values obtained in practice are much higher: 31.4 ± 2.5 (IAI-1), 14.3 ± 1.5 (IAI-2) and 6.4 ± 0.8 Ω/sq (IAI-3) (Figure 4d). Average difference is 2–8 times. The observed difference can be explained by additional scattering of charge carriers at the interfaces Ag-In_2_O_3_ and grain boundaries. The reproducibility of the synthesis of IAI structures is demonstrated in Figure S3.

For transparent conductive materials, the relationship between sheet resistance and visible transmittance is important. This criterion, called the Figure of Merit (FoM), is described in [36] and is described by the equation:

(2) $$FoM=\frac{Z_{0}}{{2R}_{s}(\frac{1}{\sqrt{T}}-1)}$$

where Z_0_—impedance of free space, equal to 377 Ω, R_s_—sheet resistance, T—transmission at a wavelength of 500 nm without taking into account the substrate. For the IAI-3 structure, the FoM value is 293.5, which is superior to ITO films on flexible substrates (Figure 4d). The complete set of optoelectronic parameters for IAI structures is given in Table 1.

It’s worth noting the extremely low scattering power of IAI structures, which is 1.04% for IAI-3. This low haze makes IAI structures promising candidates for the formation of multilayer radio-shielding and radio-absorbing assemblies.

The obtained IAI structures have an opportunity of optimization of the thickness of the In_2_O_3_ layers, with the aim of reducing the reflection coefficient and increasing the total transmittance of the multilayer film structure.

### 3.3. Spectroscopy of IAI Structures in Range 0.01–40 GHz

The mechanism of interaction of an electromagnetic wave in the radio and terahertz range, propagating in a waveguide, with a thin-film IAI structure on a dielectric substrate is: the incident wave (P_i_) is decomposed into three components: transmitted (P_t_), reflected (P_r_) and absorbed (P_a_). The corresponding coefficients can be calculated using the Formulas (3)–(5) [37].

(3) T=PtPi=10S2110·100%

(4) R=PrPi=10S1110·100%

(5) T+R+A=100%

**Figure 5:**
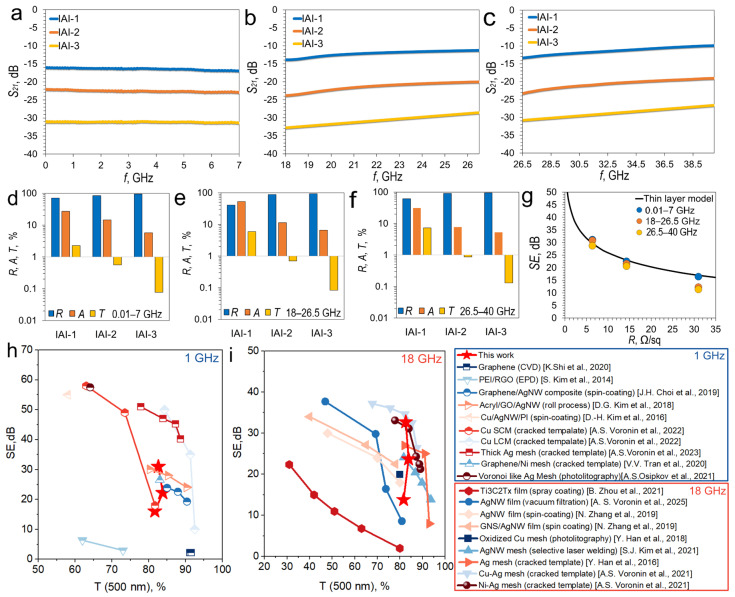
S_21_ spectra of IAI structures in the ranges—0.01–7 GHz (**a**), 18–26.5 GHz (**b**) and 26.5–40 GHz (**c**); Their averaged diagrams of the energy balance (reflection, absorbance, transmittance (R.A.T)) in the ranges—0.01–7 GHz (**d**), 18–26.5 GHz (**e**) and 26.5–40 GHz (**f**); dependence of the shielding efficiency on the value of sheet resistance (**g**). Comparison of SE indices of IAI structures with results from literature data [2,38,39,40,41,42,43,44,45] at 1 GHz (**h**) and 18 GHz (**i**).

The results of measurement S_21_ spectra of IAI structures with different Ag layer thicknesses in the range of 0.01–40 GHz are shown in Figure 5a–c.

The S_21_ of IAI structures in the frequency range of 0.01–7 GHz decreases proportionally with increasing Ag layer thickness. The S_21_ spectra are uniform and lack a pronounced frequency dependence. The average S_21_ values in this range are −16.41, −22.54, and −31.11 dB (Figure 5a).

In the high-frequency ranges of 18–40 GHz, used for 5G communications, the S_21_ also exhibits negligible frequency dependence. Due to the low S_21_ dispersion of IAI structures, their shielding properties are best described by average values. In the K-band, the average S_21_ was −12.27, −21.53 and −30.79 dB (Figure 5b), and in the K_a_-band −11.36, −20.59 and −28.69 dB (Figure 5c). Similar values in the в K- and K_a_ bands confirm the weak frequency dependence of the S_21_ he obtained data on the amplitude and frequency dependence of the S_21_ are in good agreement with the results presented in the literature [18].

The energy balance estimation based on Equations (3)–(5) is important for the analysis of shielding mechanisms. In the 0.01–7 GHz range, the IAI-1 structure with an Ag layer thickness of 7.3 ± 0.7 nm is characterized by a transmittance of 2.3%, a reflectivity of 70.6%, and an absorption coefficient of 27.1%, which indicates the predominance of the reflection mechanism (Figure 5d). Increasing the Ag thickness to 10.1 ± 0.9 nm (IAI-2) and 13.2 ± 1.1 nm (IAI-3) leads to an increase in the reflectivity to 84.9% and 94.3%, respectively. Similar trends are observed in the 18–26.5 GHz (Figure 5e) and 26.5–40 GHz (Figure 5f) ranges. For example, for the IAI-3 structure, the reflection coefficient remains high and stable in all studied ranges: 94.3% (0.01–7 GHz), 93.3% (18–26.5 GHz) and 94.6% (26.5–40 GHz).

The observed decrease in transmission and the increase reflection coefficient with increasing Ag thickness is directly correlated with the reduction in sheet resistance. The lower sheet resistance enhances the impedance mismatch between free space and the IAI structure, thereby promoting reflection as the dominant shielding mechanism. The discrepancy between the theoretical and experimental sheet resistance values, attributed to interface and grain boundary scattering (as discussed in Section 3.2), further confirms that the Ag layer’s microstructure and its interfaces with In_2_O_3_ critically influence the charge transport and, consequently, the reflection-dominated EMI shielding performance.

The IAI structure, with an Ag layer thickness of 13.2 ± 1.1 nm, has dual functionality. First, it serves as a highly effective screen, providing a shielding efficiency of approximately 30 dB (corresponding to a power attenuation of 99.9%) over a wide frequency range from 0.01 to 40 GHz. Second, this structure can function as an optically transparent broadband reflector, suitable for compensating for the attenuation and scattering of shortwave signals in urban environments, which is important for the deployment of 5G networks [46].

According to the thin layer model [47], the shielding efficiency (SE) depends on one variable: the sheet resistance of the thin film, this dependence and described by equation:

(6) $$SE=20lg(1+\frac{Z_{0}}{R_{s}})$$

where R_s_—sheet resistance of IAI structure, Z_0_ = 377 Ω—impedance of free space. Figure 5f shows an approximation of the experimental data for the dependence of the shielding efficiency, averaged over each of the three studied ranges, on the sheet resistance IAI of the structures. IAI-2 and IAI-3 structures have good agreement with the thin conductive layer model is observed in all studied ranges.

To more clearly demonstrate the high SE value and the broadband effect for IAI structures, we compare the dependences of SE on T (500 nm) at two discrete frequencies—1 GHz (Figure 5h) and 18 GHz (Figure 5i). Thus, we considered a comparison with graphene [38], PEI/rGO film [39], as well as AgNW-based composite films, such as graphene-urethane/AgNW composite [40], Acryl/GO/AgNW composite [41], Cu-AgNW/PI films [42]. We also considered materials with the highest SE values, such as highly conductive metal meshes, among which we selected: galvanic Cu meshes with large and small cells [43], thick Ag meshes [44], composite graphene/Ni mesh [45] and Voronoi-like Ag mesh [2]. Obviously, at low frequencies—1 GHz, a large number of literature results exceed the SE parameters of our IAI structures. In particular, metal meshes exhibit a very pronounced frequency dependence of SE, especially for large cell sizes [43]. Therefore, comparisons across different frequency ranges are crucial.

Comparative dependences at a frequency of 18 GHz are shown in Figure 5i. We compared the SE value with T (500 nm) at a discrete frequency of 18 GHz for the following solutions described in the literature: Ti2C2Tx films [48], Ag NW film [8,49], and GNS/AgNW composite films. Metal meshes include oxidized Cu mesh [50], AgNW mesh [51], Ag mesh [52], Cu-Ag [53], and Ni-Ag [53] meshes based on a cracked template. It is worth noting that at a frequency of 18 GHz, the balance of forces begins to shift, since at high frequencies (18 GHz), the shielding efficiency of metal meshes begins to decrease. Our IAI structures are already among the best results in the literature. We encountered certain difficulties in finding literature for a comparative analysis. This further highlights the importance of broadband measurements, which are currently quite rare; researchers generally limit themselves to the X-band (8–12 GHz).

### 3.4. THz-TDS of IAI Structures

Previous studies have extensively employed THz-TDS spectroscopy method to investigate various transparent conductors such as TCO—ITO [54], AZO and GZO [55], La-doped BaSnO_3_ [56]; carbon nanomaterials—graphene [57], MXenes [58,59] and metal structures OMO [18] AgNW [60,61].

**Figure 6:**
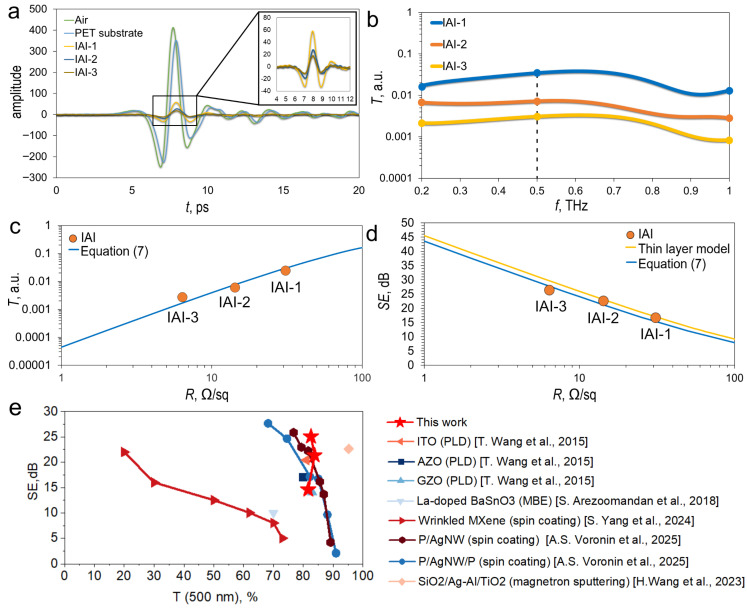
Time waveforms of THz pulses for IAI structures (**a**); Transmittance of IAI structures with different Ag thickness in the range of 0.2–1 THz (**b**); Approximation of average transmittance values in the range of 0.2–1 THz, for IAI structures with different Ag thickness, Equation (7) (**c**); Approximation of average shielding efficiency values calculated with Equation (7) (**c**) in the range of 0.2–1 THz, for IAI structures with different Ag thickness; values calculated with thin layer model and Equation (7) in the range of 0.2–1 THz, for IAI structures with different Ag thickness recalculated in SE (**d**). Comparison of SE indices of IAI structures with results from literature data [27,55,56,58,60] in the THz range (**e**). All this data obtained before mechanical tests.

We investigate the interaction of THz pulses with IAI structures based on this literature. Figure 6a shows the time-domain spectra of THz pulses transmitted through various IAI structures. The PET substrate reduces the amplitude of the transmitted THz pulse relative to empty space. A phase shift is observed, which is associated with the polarization of the substrate. Its magnitude is related to the substrate thickness by the equation: $\varphi =\frac{2\pi f}{c}nd$, where *nd*—optical path length, *f*—frequency, *c*—the speed of light in a free space. The inset shows the time signatures of THz pulses transmitted through various IAI structures with varying Ag thicknesses, in a magnified format. The graph shows that the THz pulse amplitude decays proportionally with increasing Ag layer thickness.

Figure 6b shows the transmission spectra for IAI structures in the 0.2–1 THz range. As in the 0.01–40 GHz range, the transmission spectra of IAI structures are uniform, without resonant features. The transmittance is determined by the Ag layer thickness; the thicker the layer, the lower the transmittance. The average transmittance values in the 0.2–1 THz range are –16.31, –22.92, and –26.73 dB, respectively.

The accuracy of the obtained results can be assessed by comparing the experimental results with model representations. For example, an impedance model is used to simulate the transmittance of thin films in the THz range [62]. The transmittance of thin films in the THz range can be estimated according to the equation

(7) Tf=EIAI+PET(f)EPET(f)2=1+n1+n+Z0σ(f)d2=1+n1+n+Z0/Rs2

where $E_{PET}(f)$ and $E_{IAI+PET}(f)$—the amplitudes of the electric field of the THz wave passing through the PET substrate and the PET substrate with IAI structure, σ(f)-frequency dependence of the conductivity of the IAI structure, d-thickness of Ag film, Z_0_-impedance of free space is 377 Ω, n = 1.57 refractive index of PET substrate. In the THz range, the frequency-dependent conductivity can be replaced by a static value and the following replacement can be adopted: R_s_ = 1/σ(f)d. Using Equation (7) one can approximate the experimentally obtained values of the transmittance coefficient. Figure 6c shows an approximation of the transmittance values of IAI structures with varying Ag layer thicknesses, averaged over the 0.2–1 THz range, using Equation (7). Good agreement between the experimental results and the simplest model is observed.

Figure 6d shows a comparison of the shielding efficiency (SE) with the continuous layer model described by Equation (6). Also, the calculated data obtained using Equation (7) can be converted to SE using the equation:

(8) SE=−T

The curves calculated using Equations (6) and (7) have a similar appearance and are in fairly good agreement with the average values of the shielding efficiency for IAI structures, which can be seen from a comparison of experimental data and model representations.

A comparative analysis of the SE versus T (500 nm) for a discrete frequency of 0.5 THz for our IAI structures and other transparent conductors is presented in Figure 6e. The graph shows that our IAI structures outperform classical TCO such as AZO, GZO [55], La-doped BaSnO3 [56] and promising wrinkled MXenes [58]. Composite films P/AgNW and P/AgNW/P [60] demonstrate similar parameters; however, it is worth considering that P/AgNW and P/AgNW/P films have a relatively high Haze parameter compared to IAI structures.

### 3.5. Mechanical, Chemical and Thermal Stability of IAI Structures

The main problem of transparent conductive oxides [63] is their low resistance to bending deformations, which makes them unsatisfactory materials for flexible electronics due to their brittle ceramic nature and the presence of internal stresses that lead to the formation and propagation of cracks even at small deformations.

**Figure 7:**
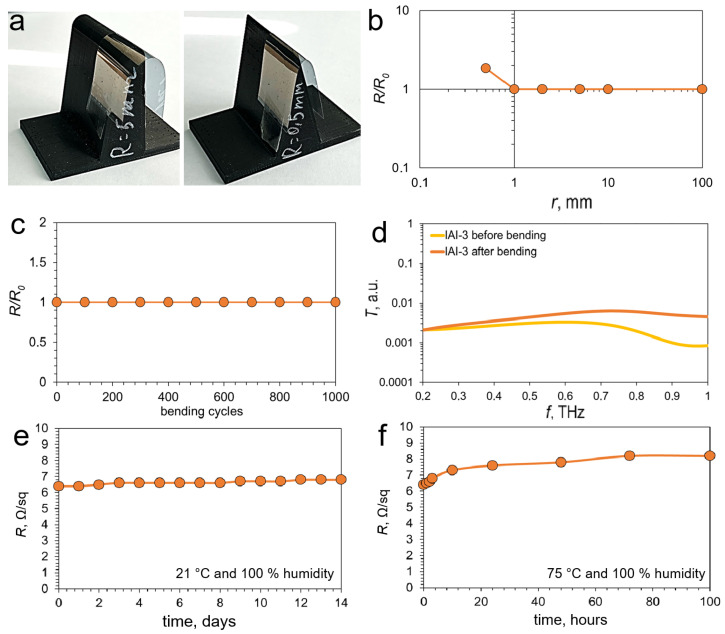
Photos of IAI-3 structure samples with different bending radii of 5 and 0.5 mm (**a**); Effect of bending radius (**b**) and cyclic bending with a radius of 5 mm (**c**) on the sheet resistance of the IAI-3 structure; Effect of cyclic bending with a radius of 5 mm of the IAI-3 structure on transmission in the range of 0.2–1 THz (**d**); Chemical stability IAI-3 structure at first mode (**e**) and second mode (**f**).

To study the resistivity of IAI structures to bending deformations, we considered the effect of a single bending with radius—100, 10, 5, 2, 1 and 0.5 mm (Figure 7a) on their sheet resistance. Figure 7b shows the dependence of the relative sheet resistance of the IAI-3 structure on the bending radius. Changes in the sheet resistance are observed only at a bending radius of 0.5 mm, with the sheet resistance increasing by 84% from 6.4 ± 0.8 Ω/sq to 11.8 ± 1.4 Ω/sq. A single bend of a larger radius did not demonstrate a significant increase in sheet resistance. The outstanding mechanical stability of IAI structures under bending is explained by the synergistic effect of the multilayer architecture. While homogeneous brittle films (e.g., TCO) are prone to the formation of through-cracks under deformation, leading to failure. In the IAI structure, the interfaces between the layers effectively suppress crack propagation. The ductile intermediate silver layer acts as a stress absorber, deflecting and arresting cracks that initiate in the brittle oxide layers. Moreover, even if cracking occurs in one of the oxide layers, the continuous conductive Ag layer and the second oxide layer maintain the overall electrical conductivity of the structure. This interface-mediated mechanism is confirmed by our experimental data: only under extreme bending (radius 0.5 mm) does significant degradation occur.

The study of fatigue stress accumulation in the IAI-3 structure is shown in Figure 7c. The structure was subjected to 1000 bending cycles with a 5 mm radius. As can be seen from the graph, the sheet resistance of the IAI-3 structure does not increase during this test.

We also investigated the effect of 1000 cyclic bends with a 5 mm radius on the THz spectra of IAI-3 structures (Figure 7d). After the bending test, a change in the spectrum shape was observed. While the transmission spectra in the 0.2–0.5 THz range remained virtually unchanged, a significant difference emerged at higher frequencies (0.5–1 THz). This discrepancy increased with frequency, reaching a maximum of 7.3 dB at 1 THz. This degradation of shielding performance at higher THz frequencies is quantitatively sup-ported by the observed microstructural changes. SEM analysis confirmed the formation of microcracks after 1000 bending cycles (Figure S4). These defects act as scattering centers, disproportionately affecting the transmission at higher frequencies where the skin depth is smaller and the integrity of the conductive layer and its interfaces becomes more critical.

Chemical stability is also a critical criterion. We investigated the resistance of the IAI-3 structure to oxidation by atmospheric oxygen and water vapor. In the first testing regime, the change in sheet resistance was monitored over two weeks at room temperature and 100% relative humidity (Figure 7e). The observations revealed a slight increase in sheet resistance from 6.4 ± 0.8 Ω/sq to 6.8 ± 0.8 Ω/sq. No visual changes were detected on the IAI-3 structure. The second testing regime, at 75 °C and 100% humidity, demonstrated a significantly greater impact on the sheet resistance (Figure 7f). Over 100 h of observation, the sheet resistance increased from 6.4 ± 0.8 Ω/sq to 8.2 ± 1.1 Ω/sq. Furthermore, characteristic point defects appeared on the sample (Figure S5). This indicates that the degradation mechanism of the IAI structures occurs via the formation of point defects, given that the indium oxide film itself acts as a substantial barrier against water vapor and atmospheric oxygen.

**Figure 8:**
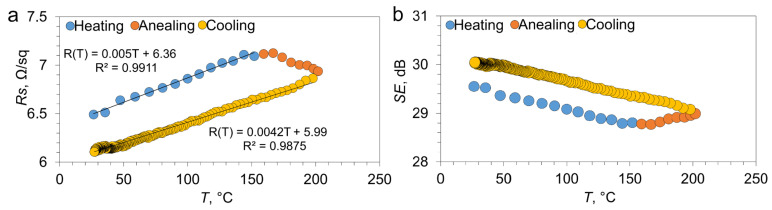
Temperature dependence of sheet resistance (**a**) and calculated SE value (**b**) for IAI-3.

It’s interesting to evaluate the influence of temperature on sheet resistance and SE. Shielded objects can heat up during operation, which in turn can lead to an increase in sheet resistance and, consequently, a change in the shielding properties of the material. To analyze this effect, we propose estimating the temperature dependence of the resistance of the IAI-3 structure and then, using the thin-layer model (Equation (6)), assessing how heating affects SE. Figure 8a shows the temperature dependence of sheet resistance for IAI-3 in the temperature range of 25–200 °C.

Based on the linear approximation of the heating curve in the temperature range of 25–150 °C (in which no changes in the transport properties of the IAI-3 structure are observed), the equation R(T) = 0.005T + 6.36 was obtained. The temperature coefficient of resistance (TCR) can be estimated from this equation:

(9) $$\alpha_{heat}=\frac{k}{R_{0}}={7.7\cdot 10}^{-4}\;{{}^{\circ}C}^{-1}$$

where k is the slope of the line, and R_0_ is the sheet resistance of the IAI structure at a temperature of 25 °C. In the temperature range of 150–200 °C, a temperature behavior of sheet resistance that is atypical for metal is observed; this is due to the annealing effect described in the literature [64]. Analysis of the cooling line as a result of the annealing process reveals a change in the coefficients in the approximation line R(T) = 0.0042T + 5.99, which will affect the value of the temperature coefficient of resistance.

(10) $$\;\alpha_{cool}=\frac{k}{R_{0}}={6.8\cdot 10}^{-4}\;{{}^{\circ}C}^{-1}$$

We obtained two TCR values: before annealing it was equal to 7.7 × 10^−4^ °C^−1^, and after the annealing operation it was equal to 6.8 × 10^−4^ °C^−1^. The tabulated value for the temperature coefficient of resistance for silver is 3.8 × 10^−3^ °C^−1^. The experimentally obtained TCR value turned out to be 4–5 times smaller than the tabulated value. This is due to the fact that the contribution of electron scattering at boundaries and defects in thin films is greater than in the bulk material. A decrease in TCR after annealing (from 7.7 × 10^−4^ °C^−1^ to 6.8 × 10^−4^ °C^−1^) indicates structural changes. A decrease in TCR may indicate an increase in grain size (recrystallization). As we have previously shown, the SE values for IAI structures averaged over different ranges are in good agreement with the thin layer model (Equation (6)). Having obtained the temperature dependence of sheet resistance, we can calculate the change in SE. The dependence of the calculated SE values on temperature for the IAI-3 structure is shown in Figure 8b. As can be seen, the dependence of SE on temperature is inversely proportional to the sheet resistance of the structure. In the temperature range of 25–150 °C, an increase in sheet resistance is observed from 6.4 ± 0.8 Ω/sq to 7.1 ± 1.1 Ω/sq, while SE decreases from 29.55 dB to 28.81 dB. After annealing and cooling the IAI-3 structure, a decrease in sheet resistance to 6.1 ± 0.6 Ω/sq is observed, while the calculated SE value increases to 30.03 dB. Due to the lower TCR relative to bulk metal, IAI structures can operate more efficiently at elevated temperatures, while the difference in SE does not exceed 1 dB in the temperature range of 25–150 °C, which has not previously been noted in the literature.

## 4. Conclusions

In this work, a series of IAI structures on a flexible PET substrate were synthesized. It has been shown that indium oxide obtained by reactive sputtering exhibits good wettability with silver; already at a silver thickness of 7.3 ± 0.7 nm, a continuous nanocrystalline film is formed. For IAI-3 structures with an Ag thickness of 13.2 ± 1.1 nm, the following optoelectronic parameters were obtained at a wavelength of 500 nm: transmittance of 82.59%, sheet resistance of 6.4 ± 0.8 Ω/sq, haze of 1.04%. Broadband measurements (10 MHz–1 THz) of the shielding efficiency of structures IAI-3 revealed a stable value within 25–30 dB over the entire range. The TCR was measured and found to be 7.7 × 10^−4^ °C^−1^, which is 4 times lower than the TCR of bulk silver. The low TCR contributes to the stabilization of the shielding properties of IAI structures in the temperature range of 25–150 °C. Low scattering ability allows us to consider In_2_O_3_/Ag/In_2_O_3_ structures and other OMO structures as promising materials for multilayer radio-shielding and radio-absorbing structures, in particular, Jauman absorbers. The results obtained in the work demonstrate the combination of excellent optoelectronic parameters in IAI structures, combined with an ultra-wideband shielding effect, which makes these materials promising for practical application.

## Figures and Tables

**Table 1:** Optoelectric parameters of IAI structures with different Ag thicknesses.

OMO Type	R_s_, Ω/sq	T_IAI+PET_ (500 nm), %	T_IAI_ (500 nm), %	Haze, %	FoM
IAI-1	31.4 ± 2.5	71.46	81.65	0.83	56.1
IAI-2	14.3 ± 1.5	73.35	83.81	0.87	143.8
IAI-3	6.4 ± 0.8	72.28	82.59	1.04	293.5

## Data Availability

The original contributions presented in this study are included in the article/Supplementary Materials. Further inquiries can be directed to the corresponding authors.

## Supplementary Materials

The following supporting information can be downloaded at https://www.mdpi.com/article/10.3390/ma18235393/s1, Figure S1. XRD patterns of IAI-1 (a), IAI-2 (b) and IAI (c); Figure S2. Histograms of sheet resistance distribution for IAI-1 (a), IAI-2 (b), and IAI-3 (c); Figure S3. Reproducibility demonstration IAI-3 structures; Figure S4. SEM images IAI-3 before (a and c) and after (b and d) 1000 bending cycles with 5 mm at different magnifications; Figure S5. Photo of IAI-3 structures after 100 h at 75 °C and 100% humidity.

## Abbreviations

**OMO**	Oxide/metal/oxide
**IAI**	In_2_O_3_/Ag/In_2_O_3_
**TCO**	Transparent conductive oxide
**SEM**	Scanning Electron Microscopy
**TEM**	Transmission Electron Microscopy
**AFM**	Atomic Force Microscopy
**THz-TDS**	THz- time domain spectroscopy
**rGO**	Reduced Graphene Oxide
**PEDOT:PSS**	poly(3,4-ethylenedioxythiophene): polystyrene sulfonate
**VHF**	Very High Frequency
**EHF**	Extremely High Frequency
**ITO**	Indium tin oxide
**AZO**	Aluminum zinc oxide
**GZO**	Gallium zinc oxide
