# Supplementary material for: Broadband EMI Shielding Performance in Optically Transparent Flexible In2O3/Ag/In2O3 Thin Film Structures
Source: Materials (Basel). 2025 Nov 29;18(23):5393. doi: 10.3390/ma18235393 (PMC12693410; doi:10.3390/ma18235393)
Supplement: Supplementary file 1 [file materials-18-05393-s001.zip › materials-3975527-supplementary.pdf]

Broadband EMI Shielding Performance in Optically Transparent Flexible In_2O_3/Ag/In_2O_3 Thin Film Structures

Anton S. Voronin[1,2,3,*], Sergey V. Nedelin[4,5], Nikita A. Zolotovsky[4,5], Igor A. Tambasov[5,6], Mstislav O. Makeev[1,*], Pavel A. Mikhalev[1], Bogdan A. Parshin[1], Evgenia L. Buryanskaya[1], Mikhail M. Simunin[1,2,7], Ilya V. Govorun[8,9], Ivan V. Podshivalov[4,8], Il`ya I. Bril`[1,2], Mikhail K. Khodzitskiy[10], Stas V. Khartov[2]

[1] Regional Educational and Scientific Center "Security" Bauman Moscow State Technical University, 105005 Moscow, Russia; pamikhalev@bmstu.ru (P.A.M.); parshbgal@bmstu.ru (B.A.P.); buryanskayael@bmstu.ru (E.L.B.); michanel@mail.ru (M.M.S.)

[2] Department of Molecular Electronics, Federal Research Center «Krasnoyarsk Scientific Center», Siberia Branch, Russian Academy of Sciences (FRC KSC SB RAS), 660036 Krasnoyarsk, Russia; ellaijiah@gmail.com (I.I.B.); stas_f1@list.ru (S.V.K.)

[3] School of Engineering and Construction, Siberian Federal University, 660041 Krasnoyarsk, Russia

[4] School of Engineering Physics and Radio Electronics, Siberian Federal University, 660041 Krasnoyarsk, Russia; s.v.nedelin@mail.ru (S.V.N.); nikitazolotovskiy@mail.ru (N.A.Z.); podshivalov.ivan@gmail.com (I.V.P.)

[5] LLC Research and Production Company «Spectehnauka», 660043 Krasnoyarsk, Russia; tambasov_igor@mail.ru (I.A.T.)

[6] Laboratory of Photonics of Molecular Systems, Kirensky Institute of Physics, Siberian Branch, Russian Academy of Sciences, 660036 Krasnoyarsk, Russia

[7] School of Non-Ferrous Metals and Materials Science, Siberian Federal University, 660041 Krasnoyarsk, Russia

[8] Laboratory of Electrodynamics and Microwave Electronics, Kirensky Institute of Physics, Siberian Branch, Russian Academy of Sciences, 660036 Krasnoyarsk, Russia; govorun-ilya@mail.ru (I.V.G.)

[9] Institute of Informatics and Telecommunication, Siberian State University of Science and Technology, 660037 Krasnoyarsk, Russia

[10] LLC "Terahertz Photonics", 191167 Saint-Petersburgh, Russia; khodzitskiy@yandex.ru (M.K.K.)

* Correspondence: a.voronin1988@mail.ru (A.S.V.); m.makeev@bmstu.ru (M.O.M)

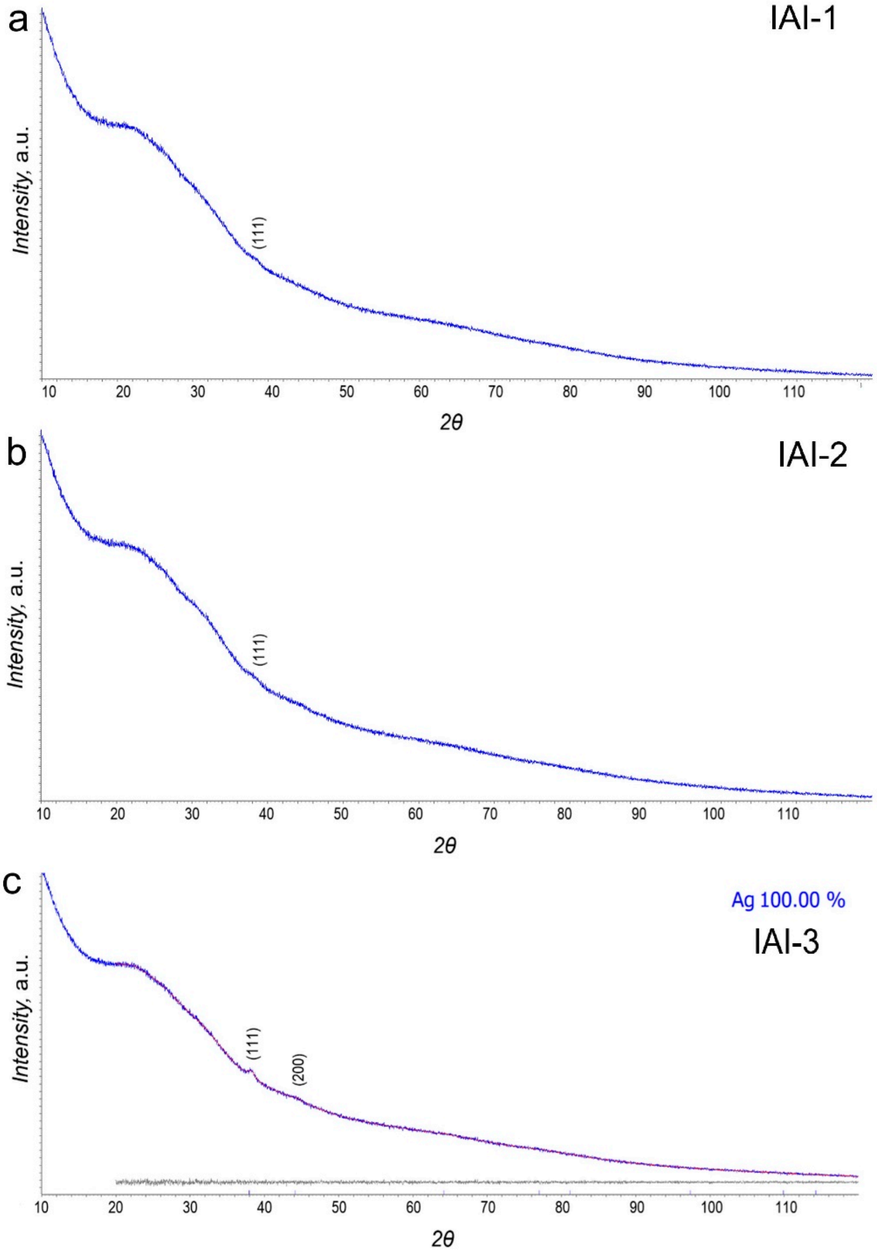

Figure S1. XRD patterns of IAI-1 (a), IAI-2 (b) and IAI (c).

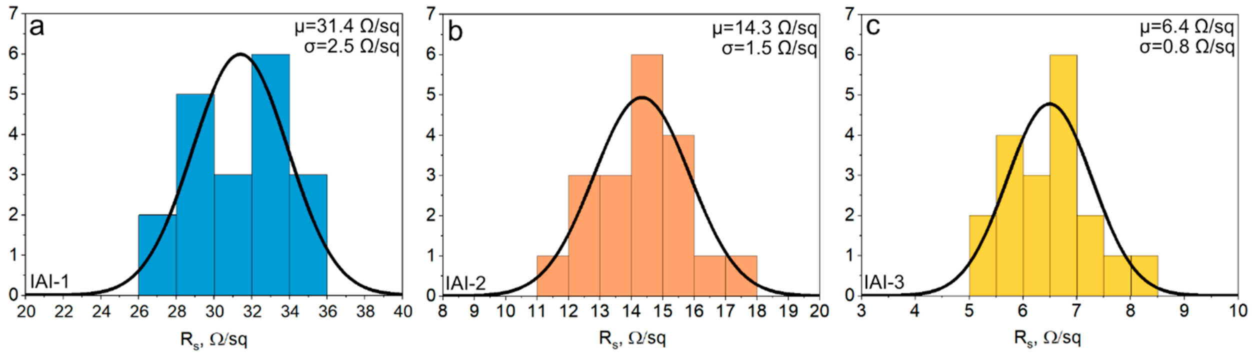

Figure S2. Histograms of sheet resistance distribution for IAI-1 (a), IAI-2 (b), and IAI-3 (c).

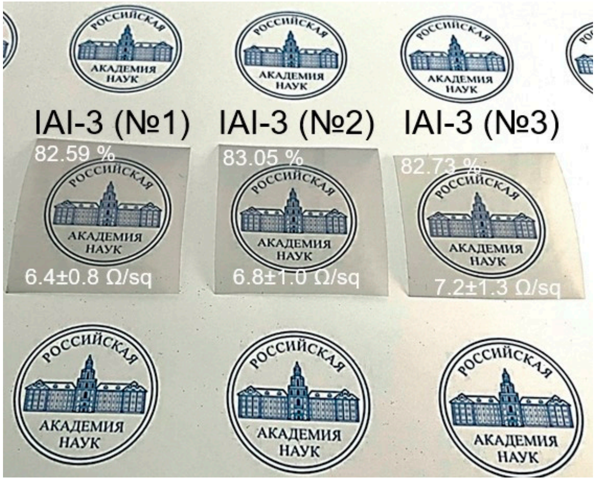

Figure S3. Reproducibility demonstration IAI-3 structures

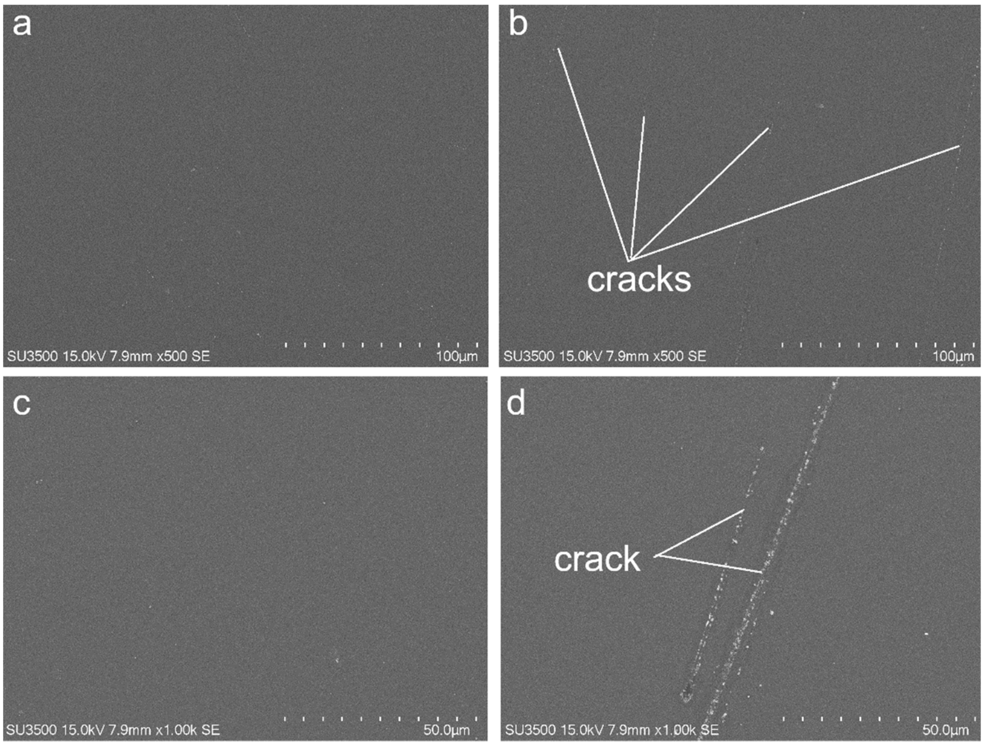

Figure S4. SEM images IAI-3 before (a and c) and after (b and d) 1000 bending cycles with 5 mm at different magnifications.

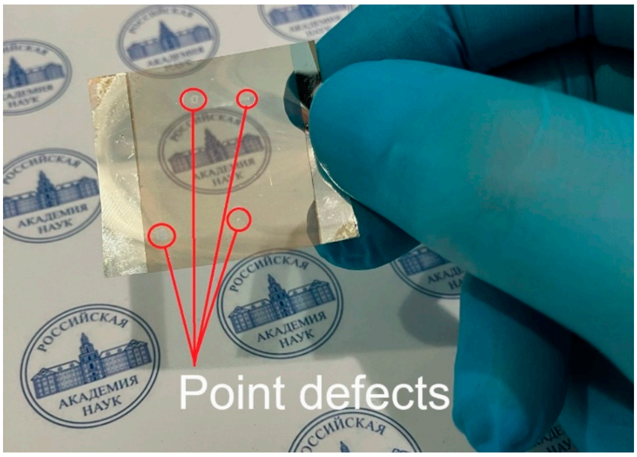

Figure S5. Photo of IAI-3 structures after 100 hours at 75 °C and 100 % humidity.
